# Genetic engineering approaches for the fermentative production of phenylglycines

**DOI:** 10.1007/s00253-020-10447-9

**Published:** 2020-02-20

**Authors:** David Moosmann, Vladislav Mokeev, Andreas Kulik, Natalie Osipenkov, Susann Kocadinc, Regina Ort-Winklbauer, Franziska Handel, Oliver Hennrich, Jung-Won Youn, Georg A. Sprenger, Yvonne Mast

**Affiliations:** 1 Microbiology/Biotechnology, Interfaculty Institute of Microbiology and Infection Medicine, Faculty of Science, University of Tübingen, Auf der Morgenstelle 28, D-72076 Tübingen, Germany; 2 Institute of Microbiology, University Stuttgart, Allmandring 31, D-70569 Stuttgart, Germany; 3 German Center for Infection Research (DZIF), Partner Site Tübingen, Tübingen, Germany; 4 Department “Bioresources for Bioeconomy and Health Research”, Leibniz Institute DSMZ-German Culture Collection for Microorganisms and Cell Cultures, 38124 Braunschweig, Germany; 5 Institute for Microbiology, Technical University of Braunschweig, 38106 Braunschweig, Germany

**Keywords:** Synthetic biology, Genetic engineering, Non-proteinogenic amino acids, D-amino acids, Phenylglycine, Actinomycetes

## Abstract

L-phenylglycine (L-Phg) is a rare non-proteinogenic amino acid, which only occurs in some natural compounds, such as the streptogramin antibiotics pristinamycin I and virginiamycin S or the bicyclic peptide antibiotic dityromycin. Industrially, more interesting than L-Phg is the enantiomeric D-Phg as it plays an important role in the fine chemical industry, where it is used as a precursor for the production of semisynthetic β-lactam antibiotics. Based on the natural L-Phg operon from *Streptomyces pristinaespiralis* and the stereo-inverting aminotransferase gene *hpgAT* from *Pseudomonas putida*, an artificial D-Phg operon was constructed. The natural L-Phg operon, as well as the artificial D-Phg operon, was heterologously expressed in different actinomycetal host strains, which led to the successful production of Phg. By rational genetic engineering of the optimal producer strains *S. pristinaespiralis* and *Streptomyces lividans*, Phg production could be improved significantly. Here, we report on the development of a synthetic biology-derived D-Phg pathway and the optimization of fermentative Phg production in actinomycetes by genetic engineering approaches. Our data illustrate a promising alternative for the production of Phgs.

**Electronic supplementary material:**

The online version of this article (10.1007/s00253-020-10447-9) contains supplementary material, which is available to authorized users.

## Introduction

To date, more than 900 naturally occurring amino acids have been identified (Lu and Freeland 2006) of which the 20 proteinogenic L-amino acids only constitute 2%. The majority of the residual 98% of non-proteinogenic amino acids serve as building blocks for bioactive natural compounds (Walsh et al. 2013). Non-proteinogenic amino acids are becoming ever more important as tools for modern drug discovery and development. On the one hand, freestanding non-proteinogenic amino acids act as antimetabolites of common amino acids and are effective inhibitors for various metabolic targets. Besides that, non-proteinogenic amino acids serve as building blocks for numerous bioactive compounds and drugs. Especially D-amino acids are of particular importance for the production of pharmaceuticals and fine chemicals. They are utilized in drugs, drug intermediates, food additives, artificial sweeteners, deodorants, insecticides, or commodity chemicals (Barredo 2005; Global Industry Analysts Inc. 2016). Annually, several tons of D-amino acids are produced. Due to an aging world population, there is a strong demand for dietary and pharmaceutical supplements, which in turn will increase the need for D-amino acids in the coming years (Global Industry Analysts Inc. 2016). One of the industrially relevant D-amino acids is D-phenylglycine (D-Phg), which is used as precursor for the production of various β-lactam antibiotics. D-Phg is a constituent of a number of semisynthetic penicillins (ampicillin, apalcillin (Boehringer Ingelheim), mezlocillin (Bayer), pivampicillin, LEO Pharma), etc.) and cephalosporins (cefalexin, cefradine, cefaclor, cefaloglycine, etc.) (Stevenazzi et al. 2014; Shiau et al. 2005; Schmid et al. 2001; Müller et al. 2013). Currently, D-Phg is produced in a scale > 5000 tons per year worldwide (Vedha-Peters et al. 2006). Until now, the amino acid is synthesized by classical or enzymatic resolution of a racemic mixture (Wegman et al. 2001). This production process is based on petrochemical feedstocks. The disadvantage of such a conventional method is that it includes many individual processing steps, which make the entire production process commercially less attractive. Besides that, chemical syntheses often need numerous chemicals and solvents, are energetically unfavorable, and/or produce a lot of waste substances. It would therefore be highly desirable to avail a more environmentally friendly fermentative route for the production of the unnatural amino acid D-Phg in future. With a fermentative production process, the substance of interest is produced by microorganisms obtained from renewable raw materials, such as glucose, whereby the end-products are characterized by a high chemo-, regio-, and stereo-selectivity. In terms of Phg, fermentative production was hampered by the fact that the amino acid is not accessible by any natural biosynthetic pathway from microbes or other organisms. In a previous approach, an artificial D-Phg production pathway has been designed in *E. coli* that applies three different enzymes from three different organisms (HmaS-hydroxymandelate synthase from *Amycolatopsis orientalis*, Hmo-hydroxymandelate oxidase from *Streptomyces coelicolor* and HpgAT–D-(4-hydroxy)phenylglycine aminotransferase from *Pseudomonas putida*), which led to the successful production of D-Phg (Müller et al. 2006). Only recently, the first natural Phg biosynthetic pathway has been reported for the antibiotic producer *Streptomyces pristinaespiralis* (Mast et al. 2011a; Osipenkov et al. 2018), which can now serve as the basis for the development of a fermentative D-Phg production route.

**Fig. 1:**
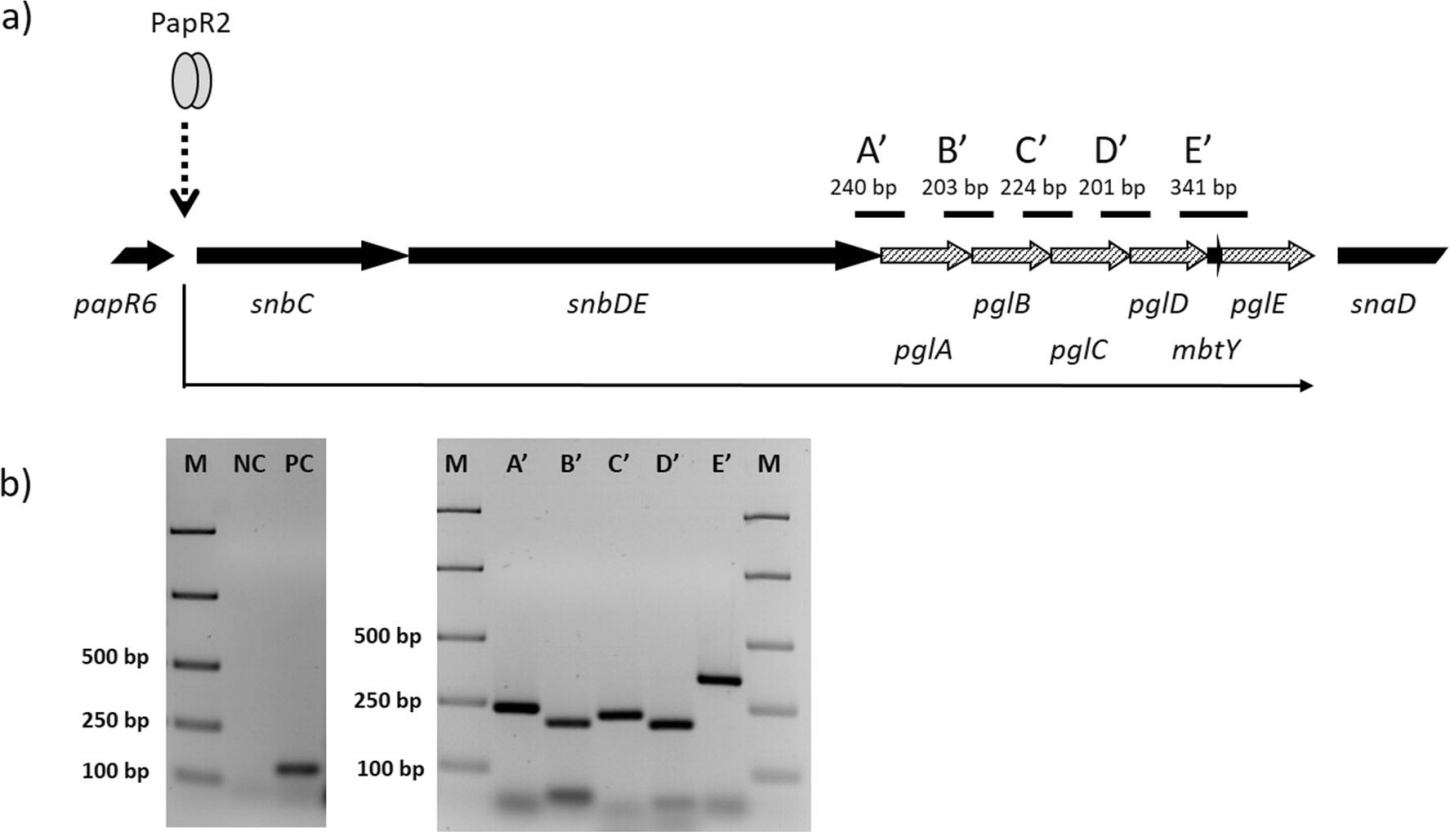
Schematic presentation of the *lpg* operon. L-Phg biosynthesis genes are shown as dashed arrows and adjacent genes as black arrows. Predicted RT-PCR amplificates (A′–E′) are shown as black lines. Transcriptional activation by PapR2 (gray ellipses) is indicated as broken arrow. Thin black arrow depicts co-transcription of genes (**a**). Transcriptional analysis of the *lpg* operon in *S. pristinaespiralis*. Total RNA was harvested after 24 h. Left figure shows RT-PCR results with *hrdB-*specific primers and total RNA (negative control, NC) and cDNA (positive control, PC) as template, respectively. Right figure shows RT-PCR results from amplification of *pgl* gene overlapping regions (amplificate A′, B′, C′, D′, and E′, respectively). A total of 5 μl of the 1 kb ladder from Fermentas was used as an internal standard (M) (**b**)

*S. pristinaespiralis* is the producer of the streptogramin antibiotic pristinamycin, which consists of the two chemically non-related substances pristinamycin I (PI) and pristinamycin II (PII). PI is synthesized by the nonribosomal peptide synthetases (NRPSs) SnbA, SnbC, and SnbDE, whereby the latter one incorporates L-Phg as the final amino acid into the growing PI peptide chain (Mast et al. 2011b; Mast and Wohlleben 2014). Within the pristinamycin biosynthetic gene region, the genes *pglA*, *pglB*, *pglC*, *pglD*, and *pglE* are organized in an operon-like structure (*lpg*) and together encode for L-Phg biosynthesis (Mast et al. 2011a, 2015, Osipenkov 2016). These genes are located downstream of the NRPS genes *snbC* and *snbDE* and are under control of the pathway-specific transcriptional activator PapR2 (Mast et al. 2015) (Fig. 1a).

**Fig. 2:**
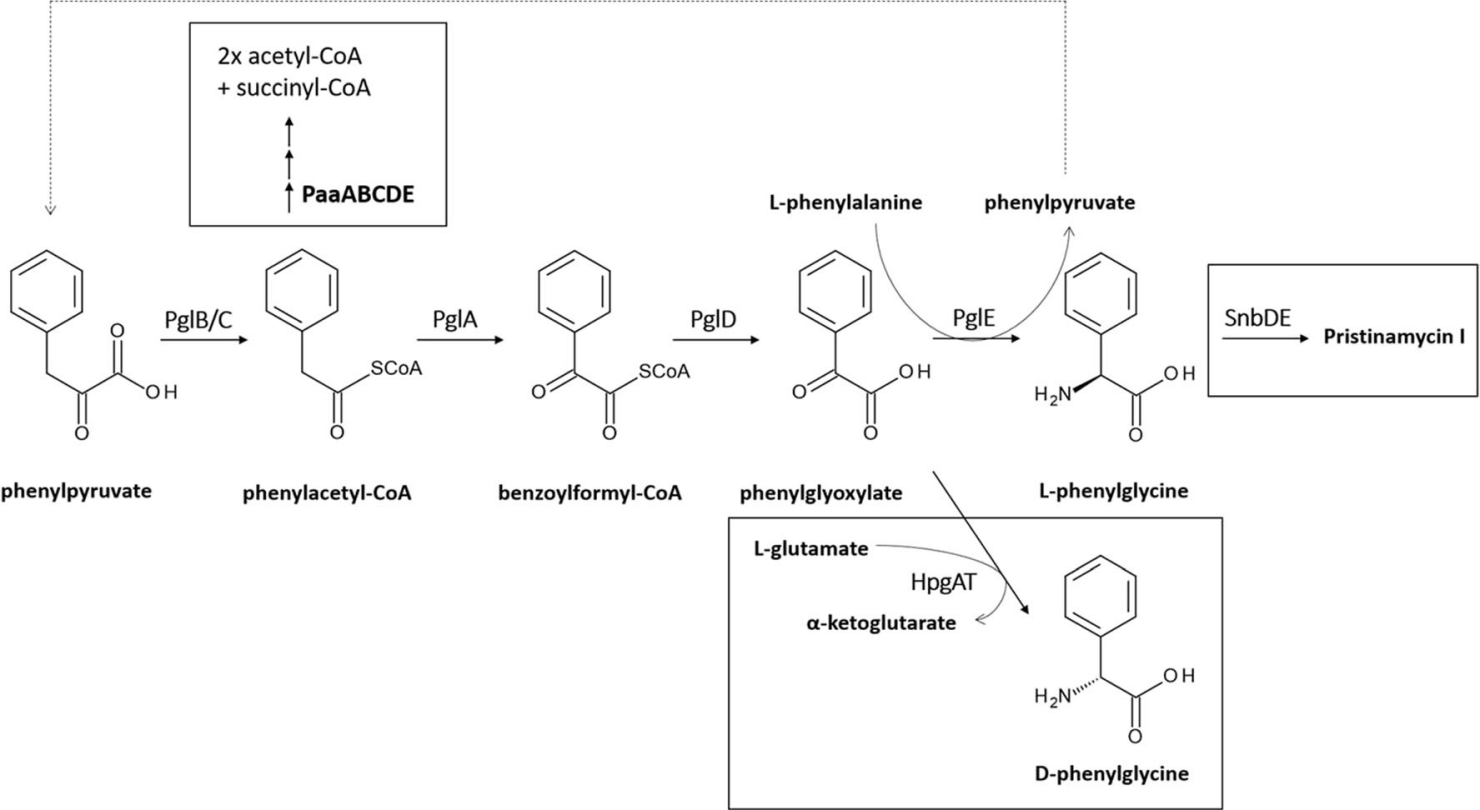
Schematic presentation of the natural L-Phg biosynthetic pathway from *S. pristinaespiralis.* Biochemical reactions targeted by genetic engineering, such as the HpgAT catalyzed reaction resulting in D-Phg production, PaaABCDE-catalyzed Phenylacetyl-CoA degradation, and SnbDE-catalyzed incorporation of L-Phg into PI, are highlighted in black boxes

L-Phg in *S. pristinaespiralis* is suggested to originate from the shikimate pathway. As a first metabolic step, phenylpyruvate is converted to phenylacetyl-CoA by the action of a pyruvate dehydrogenase–like complex PglB/C. Phenylacetyl-CoA is oxidized to benzoylformyl-CoA via the Phg dioxygenase PglA. The CoA residue from benzoylformyl-CoA is cleaved off by the thioesterase PglD, resulting in the formation of phenylglyoxylate. In a final reaction step, phenylglyoxylate is converted to L-Phg by the aminotransferase PglE (Mast et al. 2011b). As PglE uses L-phenylalanine as amino group donor for the transamination reaction, phenylpyruvate is formed as the α-keto acid product, which can re-enter Phg biosynthesis as a precursor (Osipenkov et al. 2018) (see Fig. 2).

L-Phg is a rare amino acid, which only occurs in a few natural products, such as the related streptogramin antibiotic virginiamycin S from *Streptomyces virginiae* (Ningsih et al. 2011) or the bicyclic peptide antibiotics dityromycin, produced by *Streptomyces* sp. strain AM-2504; GE82832 of *Streptosporangium cinnabarinum* strain GE82832, or MBJ-0086 and MBJ-0087, isolated from *Sphaerisporangium* sp. 3226 (Al Toma et al. 2015). There is also an industrial demand for L-Phg since it is used as a component of the synthetic cyclic hexadepsipeptide pasireotide (Signifor®, Novartis), which is a somatostatin analogue used for the treatment of Cushing’s disease. L-Phg is also used for the synthesis of the antitumor compound taxol (Croteau et al. 2006; Denis et al. 1991; Wang et al. 1994) and the synthesis of DAPT (N-[N-(3,5-difluorophenacetyl)-L-alanyl]-S-*phenylglycine* t-butyl ester), which acts as an inhibitor of the human *γ-secretase, a target used for the treatment of Alzheimer’s disease and different types of cancer* (Kan et al. 2004). Besides its application for diverse pharmaceuticals, L-Phg, as well as the enantiomeric D-Phg, can be used for the synthesis of the artificial non-nutritive sweetener aspartame (Ebeling 1998; Janusz 1986; Schutt 1981).

In this study, we describe the development of a synthetic biology-derived D-Phg pathway. Furthermore, we report on genetic engineering approaches in order to optimize Phg production in actinomycetal expression strains.

## Material and methods

### Bacterial strains, plasmids, and cultivation conditions

Bacterial strains, plasmids, cosmids, and primers used in this study are listed in Table S1. An overview of genes used for this study is given in Table 1. For routine cloning strategies, *Escherichia coli* XL1-Blue was used. *E. coli* strains were grown in Luria-Bertani (LB) medium at 37 °C (Sambrook et al. 1989) supplemented with kanamycin or apramycin (50 or 100 μg/ml, respectively) when appropriate. For cultivation and harvesting of genomic DNA, *Streptomyces* strains were grown in 100 ml of S-medium (Kieser et al. 2000) in 500-ml Erlenmeyer flasks (with steel springs) on an orbital shaker (180 rpm) at 28 °C. For pristinamycin production analyses, cells were grown and treated as reported previously (Mast et al. 2011a).

**Table 1:** List of genes mentioned in this study with encoded functions

Gene	Origin	Function
*pglA*	*S. pristinaespiralis*	Phenylglycine dehydrogenase
*pglB*	*S. pristinaespiralis*	Pyruvate dehydrogenase α-subunit
*pglC*	*S. pristinaespiralis*	Pyruvate dehydrogenase β-subunit
*pglD*	*S. pristinaespiralis*	Thioesterase
*pglE*	*S. pristinaespiralis*	L-phenylglycine aminotransferase
*hpgAT*	*P. putida*	D-phenylglycine aminotransferase
*snbDE*	*S. pristinaespiralis*	Pristinamycin I–specific nonribosomal peptide synthetase
*papR5*	*S. pristinaespiralis*	TetR-like repressor of pristinamycin biosynthesis
*paaABCDE* (*paa* operon)	*S. pristinaespiralis*, *S. lividans*	phenylacetyl-CoA epoxidase multicomponent enzyme system

### Transcriptional analysis by RT-PCR experiments

*S. pristinaespiralis* Pr11 wild type was grown in pristinamycin inoculum and production medium as reported previously (Mast et al. 2011a). Samples were harvested after 24 h. RNA isolation and RT-PCR procedure were carried out as described before (Mast et al. 2015). For RT-PCR reactions, primers RTpglfw/rv were used that anneal to overlapping regions of the *pgl* gene sequences. As an internal control, RT-PCR was performed with primers targeting the major sigma factor transcript *hrdB*. To exclude DNA contamination, negative controls were carried out by using total RNA as a template for each RT-PCR reaction.

### Construction of Phg expression plasmids

#### *lpg* expression construct

For cloning of the native *lpg* operon from *S. pristinaespiralis*, the pYJM1 cosmid DNA, harboring the *pglA-E* genes, was used as a template in a PCR approach with the primers lpgfw/rv and KAPAHiFi™ polymerase (Peqlab). The lpgfw/rv primer pair was designed in a way that a *Nde*I (5′ end) and *Hin*dIII (3′ end) restriction sequence is added to the *lpg* amplificate. The ~ 6-kb *lpg* fragment was subcloned into the linear PCR cloning vector pJET1.2/blunt (Fermentas), which resulted in the construct pJET/lpg. *lpg* was isolated from pJET/lpg as a *Nde*I/*Hin*dIII fragment and was cloned into the *Nde*I/*Hin*dIII-restriction site of the expression vector pRM4 under control of the constitutive erythromycin resistance gene promoter, *P*_*ermE*_, resulting in the *lpg* expression construct pYM/lpg (Fig. S1).

#### *dpg* expression construct

On the basis of the natural *lpg* operon from *S. pristinaespiralis,* an artificial *dpg* operon was constructed. For this purpose, the *Nde*I/*Hin*dIII *lpg* fragment from pJET/lpg (see above) was subcloned into the *Nde*I/*Hin*dIII-restricted *E. coli* vector pK18 (Pridmore 1987), resulting in construct pK18/lpg. For cloning of the *dpg* operon, a recombinant PCR approach was conducted in order to fuse the *pglD* gene from *S. pristinaespiralis* to the *hpgAT* gene of *P. putida*. To amplify the *pglD* fragment, the pYJM1 cosmid DNA was used as a template together with primers pglDfus1/2 for PCR amplification, resulting in fragment *pglD′* with a size of ~ 800 bp. The *hpgAT* gene (accession number AX467211) from *P. putida* was synthesized de novo (Mr. Gene GmbH, Regensburg) and used as a template for PCR amplification with primers hpgATfus1/2, which resulted in the ~ 1.4-kb fragment *hpgAT′*. Primers pglDfus2 and hpgATfus1 had 20-bp complementary 5′-3′ sequences, which allowed annealing of the fragments in a recombinant PCR approach. *pglD′* and *hpgAT′* were used as templates for recombinant PCR with primers pglDfus1/hpgATfus2, resulting in the fusion product *pglD-hpgAT′* (~ 2.2 kb). *pglD-hpgAT′* was subcloned in the PCR cloning plasmid pDrive (Qiagen), which resulted in the construct pDrive/*pglD-hpgAT′*. The correctness of the gene sequence was verified by the primer walking method (GATC Biotech, Konstanz). The plasmid pK18/lpg was cleaved with *SfiI/HindIII* and the ~ 6.3-kb pK18/*pglA-pglC* fragment was ligated to the *pglD-hpgAT′* fragment, which was excised with the same restriction enzymes from pDrive/*pglD-hpgAT′*, resulting in the construct pK18/dpg. The artificial *dpg* operon was isolated from pK18/dpg as a *Nde*I/*Hin*dIII fragment and was subcloned into the *Nde*I/*Hin*dIII-restricted pRM4 plasmid, resulting in the *dpg* expression construct pYM/dpg (Fig. S1).

#### Expression constructs with thiostrepton resistance cassettes

In order to select for Phg operon containing transformants of apramycin-resistant mutants (*MpglE* (Mast et al. 2011a) and *papR5::apra (*Mast et al. 2015)), expression constructs were designed, which harbor a thiostrepton resistance cassette (*thio*^*R*^). For this purpose, the *thio*^*R*^ cassette was isolated as a *Xba*I-restricted fragment from pDrive-thio and was cloned into the *Xba*I restriction site of pRM4, pYM/lpg, and pYM/dpg, resulting in the expression constructs pYMT, pYMT/lpg, and pYMT/dpg, respectively (Fig. S1).

### Transformation of strains and culture conditions

The targeting plasmids pYM/*lpg* and pYM/*dpg* were each transferred to *S. pristinaespiralis* Pr11, *S. lividans* T7, *S. albus*, *A. balhimycina*, and *R. jostii* RHA1 by protoplast transformation (Kieser et al. 2000), resulting in the expression strains *SPlpg-OE*, *SPdpg-OE*, *SLlpg-OE*, *SLdpg-OE*, *SAlpg-OE*, *SAdpg-OE*, *ABlpg-OE*, *ABdpg-OE*, *RJlpg-OE*, and *RJdpg-OE*, respectively. Strains with the empty pRM4 vector served as control (*SP-C*, *SL-C*, *SA-C*, *AB-C*, and *RJ-C*, respectively). All strains were inoculated from R5 agar into three independent replicates of 100 ml of preculture medium (R5 or pristinamycin production medium HT7T). R5 contained (per liter) the following: sucrose 103 g; yeast extract, 5 g; glucose, 10 g; TES ([N-tris(hydroxymethyl)methyl-2-aminoethanesulfonic acid], 5.75 g; K_2_SO_4_, 0.25 g; MgCl_2_, 10.12 g; casamino acids, 0.1 g; L-proline, 3 g; KH_2_PO_4_, 0.05 g; CaCl_2_x2H_2_O, 2.94 g, 2 ml of trace elements stock solution; pH 7.4 and HT7T contained (per liter): white dextrin, 10 g; NZ amine-A, 2 g; LabLemco beef powder, 1 g; yeast extract, 1 g; 1 ml of trace elements stock solution; pH 7.4 (Kieser et al. 2000; Folcher et al. 2001). Strains were cultivated in 500-ml Erlenmeyer flasks (with steel springs) on an orbital shaker (180 rpm) at 30 °C. After 72 h, 7 ml of preculture were inoculated into 100 ml of production medium (R5 or HT7T, respectively) and the main culture was grown for 24, 30, 48, 72, or 96 h, respectively. Ten milliliters of sample was harvested and centrifuged at 5000 rpm for 10 min. One milliliter of culture filtrate was used for HPLC-MS/MS analysis.

### Construction of mutants and mutant-derived expression strains

Construction and verification of the mutants *MsnbDE::thio*, *SPpaa::thio*, and *SLpaa::thio*, as well as construction of all mutant-derived expression strains, is described in Supplementary File.

### HPLC-MS/MS analysis of phenylglycine

HPLC-MS/MS analysis has been performed as described previously (Osipenkov et al. 2018). Tandem MS (MS/MS) was carried out in the positive mode for phenylglycine (Phg) (precursor ion m/z 152) with the corresponding target mass. Phg amount was measured in counts corresponding to the peak height. Phg concentration was calculated by reference to a standard curve using suitable concentrations of pure Phg (Fluka). Data are presented as the averages of the results from three independent biological replicates.

## Results

### Phg biosynthetic genes are co-transcribed as a multi-gene operon

As described above, the L-Phg biosynthetic genes (*pglA-E*) are organized in an operon-like structure (*lpg*) within the pristinamycin biosynthetic gene region (Fig. 1a) (Mast et al. 2011b). *lpg* is localized between the genes *snbDE* and *snaD*, which encode PI- and PII-specific peptide synthetases, respectively (Mast et al. 2011a). The gene *mbtY* is embedded in the *lpg* region and encodes a MbtH-like protein, which is suggested to interact with SnbDE but is not directly involved in Phg biosynthesis (Mast et al. 2011b). In order to determine if the *pgl* genes are co-transcribed and to ensure a successful transcription of the *lpg* operon in the heterologous expression studies later on, RT-PCR experiments have been conducted with RNA isolated from the *S. pristinaespiralis* wild type and primers that anneal to overlapping regions of the *pgl* genes (Fig. 1a). With these experiments, amplicons were obtained, which are specific for the overlapping regions between *snbDE* and *pglA* (A′), *pglA* and *pglB* (B′ ), *pglB* and *pglC* (C′), *pglC* and *pglD* (D′ ), and *pglD* and *pglE* (E′ ), respectively, revealing that all *pgl* genes are transcribed as one polycistronic mRNA and form an operon together with the Phg-specific NRPS gene *snbDE* (Fig. 1b). Since *snbDE* is located directly downstream of *snbC* with overlapping stop and start codons, respectively, and *snbC* has been shown to be regulated by PapR2, it can be estimated that *snbC*, *snbDE*, and the *pgl* genes together form a multi-gene operon, which is under regulatory control of the pristinamycin pathway-specific activator PapR2 (Fig. 1a).

### Expression of L- and D-Phg operons in suitable host strains

**Fig. 3:**
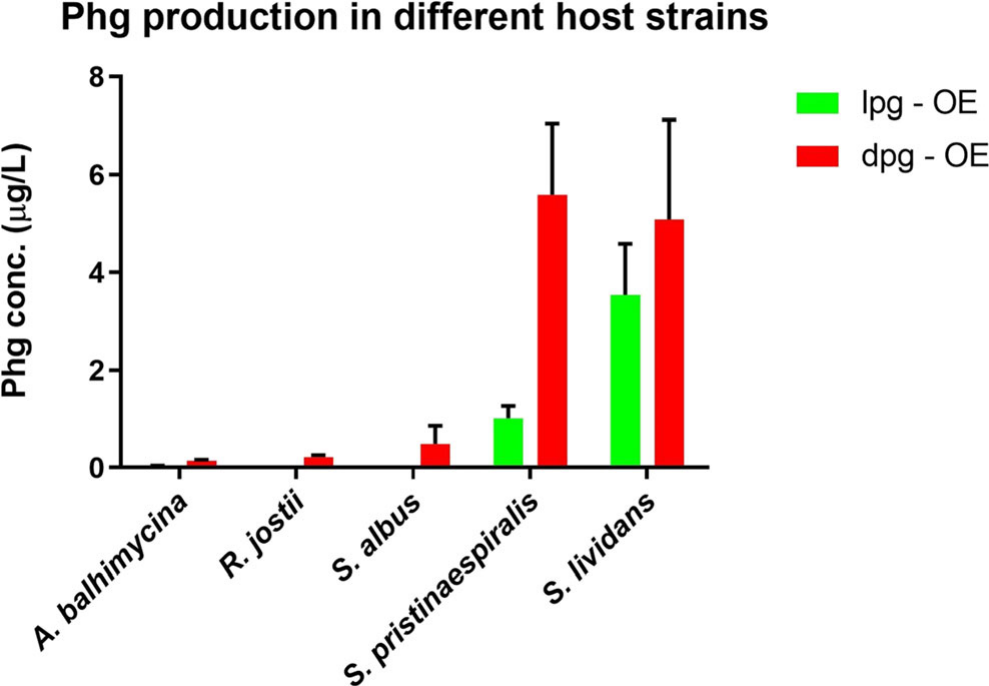
Phg production of the different expression strains *A. balhimycina* (*ABlpg-OE*, *ABdpg-OE*), *R. jostii* (*RJlpg-OE*, *RJdpg-OE*), *S. albus* (*SAlpg-OE*, *SAdpg-OE*), *S. pristinaespiralis* (*SPlpg-OE*, *SPdpg-OE*), and *S. lividans* (*SLlpg-OE*, *SLdpg-OE*) grown in R5 medium. Phg production was measured at 30 h. Phg concentration is given in micrograms per liter. Data were obtained from three independent biological replicates

To obtain constructs for the fermentative production of L-Phg, the native ~ 6-kb *lpg* operon from *S. pristinaespiralis* was cloned into the integrative vector pRM4 under control of the constitutive ermE* promoter, resulting in the expression construct pYM/lpg (Fig. S1). For production of the D-Phg enantiomer, an artificial D-Phg operon (*dpg*) was generated on the basis of the native *lpg* operon from *S. pristinaespiralis:* In a synthetic biology approach, the gene *pglE,* encoding the L-Phg aminotransferase in *S. pristinaespiralis,* was exchanged by the gene *hpgAT* from *P. putida*, which codes for a stereospecific D-Phg aminotransferase. This D-Phg aminotransferase is the only currently known L to D stereo-inverting aminotransferase (Walton et al. 2018). A recombinant PCR yielded the artificial *dpg* operon, which was cloned into pRM4, resulting in the expression construct pYM/dpg (Fig. S1). Both plasmids, pYM/lpg and pYM/dpg, were each transferred into different actinomycetes (*S. pristinaespiralis* Pr11, *Streptomyces lividans* T7, *Streptomyces albus* J1074, *Amycolatopsis balhimycina*, and *Rhodococcus jostii* RHA1) as homologous or heterologous host strains, respectively (–OE strains; Supplementary File). Strains with the empty pRM4 vector served as control (–C strains; Supplementary File). *S. pristinaespiralis* was used as expression strain because it is the natural producer of L-Phg, which is a building block for the biosynthesis of the streptogramin antibiotic PI. *A. balhimycina* was tested since it produces the structurally related non-proteinogenic amino acids hydroxy- and dihydroxy-phenylglycine, which are components of the glycopeptide antibiotic balhimycin (Pfeifer et al. 2001). *S. lividans* and *S. albus* are established heterologous expression strains (Nah et al. 2017) and *R. jostii* has a well-studied, intensive aromatic compound metabolism (Yam et al. 2011). All strains were grown in R5 medium in triplicate. After 30-h, supernatant samples were harvested and Phg amount (given in μg/L) was determined by HPLC-MS/MS analysis. Here, it should be noted that the applied method does not allow to distinguish between different Phg enantiomers. In order to determine enantiomerism of the produced Phg compounds, chiral HPLC analyses have been performed with various expression samples. However, Phg concentrations were too low to be detected (data not shown). HPLC-MS/MS analysis revealed that Phg amount was largest in samples from *S. lividans* (SL) and *S. pristinaespiralis* (SP) expression strains (> 1 μg/L), whereas only minor Phg amounts were measured for samples of *S. albus* (SA), *A. balhimycina* (AB), and *R. jostii* (RJ) expression strains (< 0.75 μg/L) (Fig. 3). No, or only trace amounts of Phg were detected in the respective pRM4 control samples (–C strains, data not shown). Interestingly, all D-Phg expression samples contained higher amounts of Phg than the respective L-Phg expression samples (Fig. 3). Altogether, from all tested strains, *S. lividans* and *S. pristinaespiralis* turned out to be the optimal hosts for fermentative Phg production.

### Optimal production media for Phg production

**Fig. 4:**
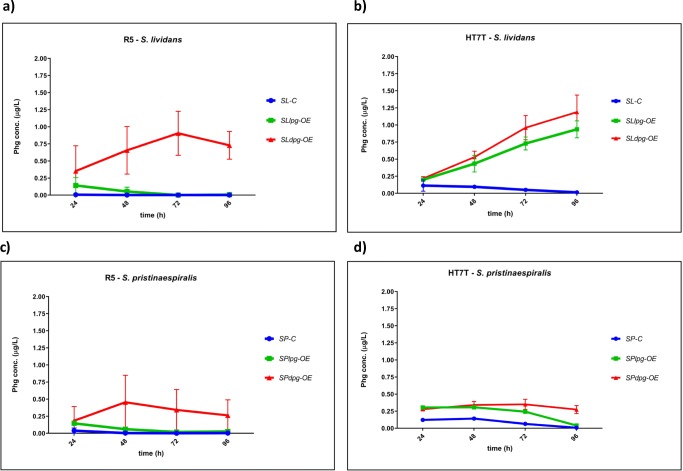
Phg production of *S. lividans* Phg expression strains *SLlpg-OE* and *SLdpg-OE*, (control: *SL-C*) in R5 (**a**) and HT7T (**b**). Phg production of *S. pristinaespiralis* Phg expression strains *SPlpg-OE* and *SPdpg-OE*, (control: *SP-C*) in R5 (**c**) and HT7T (**d**). Phg production was measured at 24, 48, 72, and 96 h. Phg concentration is given in micrograms per liter. Data were obtained from three independent biological replicates

In order to define the best Phg production conditions, the optimal producer strains *S. pristinaespiralis* (*SPlpg-OE*, *SPdpg-OE*) and *S. lividans* (*SLlpg-OE*, *SLdpg-OE*) were grown in two different culture media—the complex medium R5 and the pristinamycin production medium HT7T. Samples were taken at different time points (24, 48, 72, and 96 h) and Phg amount was determined by HPLC-MS/MS. Phg was detected in all *S. pristinaespiralis* (*SPlpg-OE*, *SPdpg-OE*) and *S. lividans* (*SLlpg-OE*, *SLdpg-OE*) expression samples, whereas only trace amounts of Phg were measured in the respective pRM4 control samples (Fig. 4a–d). Overall, Phg production was generally higher (even if statistically significant only for L-Phg expression samples as shown in Fig. S2) and more consistent in HT7T medium than in R5 (Fig. 4b, d vs a, c). Interestingly, Phg concentrations decreased in nearly all media and all expression hosts after reaching the maximal level, which suggests a degradation or metabolization of the expression product. An exception was found for *S. lividans* expression strains in HT7T medium, where Phg production steadily increased to cultivation time point 96 h (Fig. 4b). Thus, Phg metabolization in *S. lividans* seems to be medium dependent. For *S. pristinaespiralis* samples, Phg decrease in the pristinamycin production medium HT7T might also be explained by a subsequent incorporation of Phg into PI. Furthermore, it was observed that Phg concentrations in general were higher in D-Phg expression strains than in L-Phg expression strains, which was consistent with the data obtained from the Phg expression studies in different host strains (Fig. 4a–d vs Fig. 3). D-amino acids are known for their poor metabolic usability (Elmadfa and Leitzmann 2015). Hence, the higher Phg amount in the D-Phg expression strains might be explained by a rather poor metabolization of the unnatural D-Phg enantiomer. Due to the observation that overall Phg production was more stable and consistent in HT7T and with regard to subsequent genetic engineering approaches targeting pristinamycin-specific genes in *S. pristinaespiralis* host strains (see below), the pristinamycin production medium HT7T was used as Phg production medium for further analyses.

### Deletion of a gene of the phenylacetyl-CoA degradation pathway significantly improves Phg production in *S. pristinaespiralis* but not in *S. lividans*

**Fig. 5:**
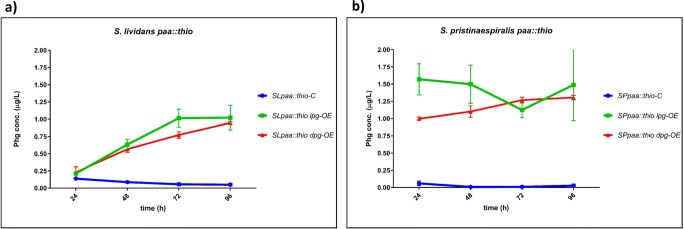
Phg production of *S. lividans paa* host strains *SLpaa::thio lpg-OE* and *SLpaa::thio dpg-OE*, (control: *SLpaa::thio-C*) (**a**) and *S. pristinaespiralis paa* host strains *SPpaa::thio lpg-OE* and *SPpaa::thio dpg-OE*, (control: *SPpaa::thio-C*) (**b**) grown in HT7T. Phg production was measured at 24, 48, 72, and 96 h. Phg concentration is given in micrograms per liter. Data were obtained from three independent biological replicates

In order to increase Phg production in the optimal producer strains *S. lividans* and *S. pristinaespiralis*, we aimed to genetically manipulate key steps within primary metabolism involved in precursor supply to direct the metabolic flux towards Phg production. As a target of manipulation, we chose the phenylacetyl-CoA degradation pathway since phenylacetyl-CoA is a suggested precursor for the biosynthesis of Phg (Mast et al. 2011a; Osipenkov et al. 2018) (Fig. 2). In a previous study from Zhao et al. (2015), it has been reported that the *paaABCDE* (*paa*) operon from *S. pristinaespiralis* encodes a putative phenylacetyl-CoA epoxidase multicomponent enzyme system, which is responsible for the degradation of phenylacetyl-CoA (Zhao et al. 2015). It was suggested that derepression of the *paa* operon in *S. pristinaespiralis* leads to a higher flux of phenylacetyl-CoA towards the phenylacetic acid catabolic pathway and thus to less precursor supply for L-Phg biosynthesis (Zhao et al. 2015). By contrast, it can be assumed that an inactivation of the *paa* genes in *S. pristinaespiralis* drives the phenylacetyl-CoA flux towards Phg biosynthesis. Thus, we aimed to inactivate the *paa* operon in *S. pristinaespiralis*—but also *S. lividans*, since a homologous *paa* operon is present in the *S. lividans* genome (Supplementary File)—and overexpress the Phg operons in the engineered mutant strains in order to increase production yields. For this purpose, the gene region *paaA-E* in *S. pristinaespiralis* and *S. lividans*, respectively, was inactivated by replacing it against a thiostrepton resistance cassette (*thio*^*R*^) (Supplementary File, Fig. S3). This resulted in the mutants *SPpaa::thio* and *SLpaa::thio*, respectively, in which the Phg expression constructs pYM/lpg and pYM/dpg, as well as the empty vector as a control, were each transferred to. The *paa* control strains, *SPpaa::thio-C* and *SLpaa::thio-C* and the host strains *SPpaa::thio lpg-OE*, *SPpaa::thio dpg-OE*, *SLpaa::thio lpg-OE*, and *SLpaa::thio dpg-OE* were grown in HT7T medium and supernatant samples at different time points were used for Phg production analysis. HPLC-MS/MS measurements of the samples from the engineered host strains revealed that Phg production in the *S. lividans paa* expression samples was almost the same as in the wild-type-derived expression samples (Fig. 5a vs Fig. 4b): maximal Phg production at 96 h was measured for *SLpaa::thio lpg-OE* at 1.00 μg/L compared with 0.94 μg/L for *SLlpg-OE* and 0.95 μg/L for *SLpaa::thio dpg-OE* compared with 1.2 μg/L for *SLdpg-OE.* In contrast, Phg production was strongly improved for *S. pristinaespiralis paa*-derived expression samples (Fig. 5b vs Fig. 4d): Already after 24 h, Phg amount in *SPpaa::thio lpg-OE* (1.57 μg/L) was 5-fold higher than in *SPlpg-OE* (0.31 μg/L) and remained high until 96 h. Here, the production decline at 72 h might be an artifact since standard deviations for the *SPpaa::thio lpg*-OE samples in general were quite high. Phg production was also significantly improved for *SPpaa::thio dpg-OE* strains, where the maximal Phg production at 96 h (1.30 μg/L) was 3.7-fold higher than in non-engineered *SPdpg-OE* strains (0.35 μg/L). Overall, the significant improvement of Phg production in *S. pristinaespiralis paa* host strains most likely results from the directed flux of the phenylacetyl-CoA precursor towards the Phg biosynthetic pathway. The fact that Phg production was improved for *SPpaa::thio lpg*-OE compared with *SPpaa::thio dpg*-OE might be explained by the different enzyme kinetics of the two aminotransferases. D-amino acid transaminases, such as HpgAT (encoded in the *dpg* operon), are commonly known to have a very low transamination activity towards D-Phg (Soda and Esaki 1994). Thus, PglE may convert the accruing phenylglyoxylate precursor more efficiently to L-Phg than HpgAT can convert it to D-Phg.

### Deletion of Phg aminotransferase gene *pglE* slightly improves Phg production in *S. pristinaespiralis*

**Fig. 6:**
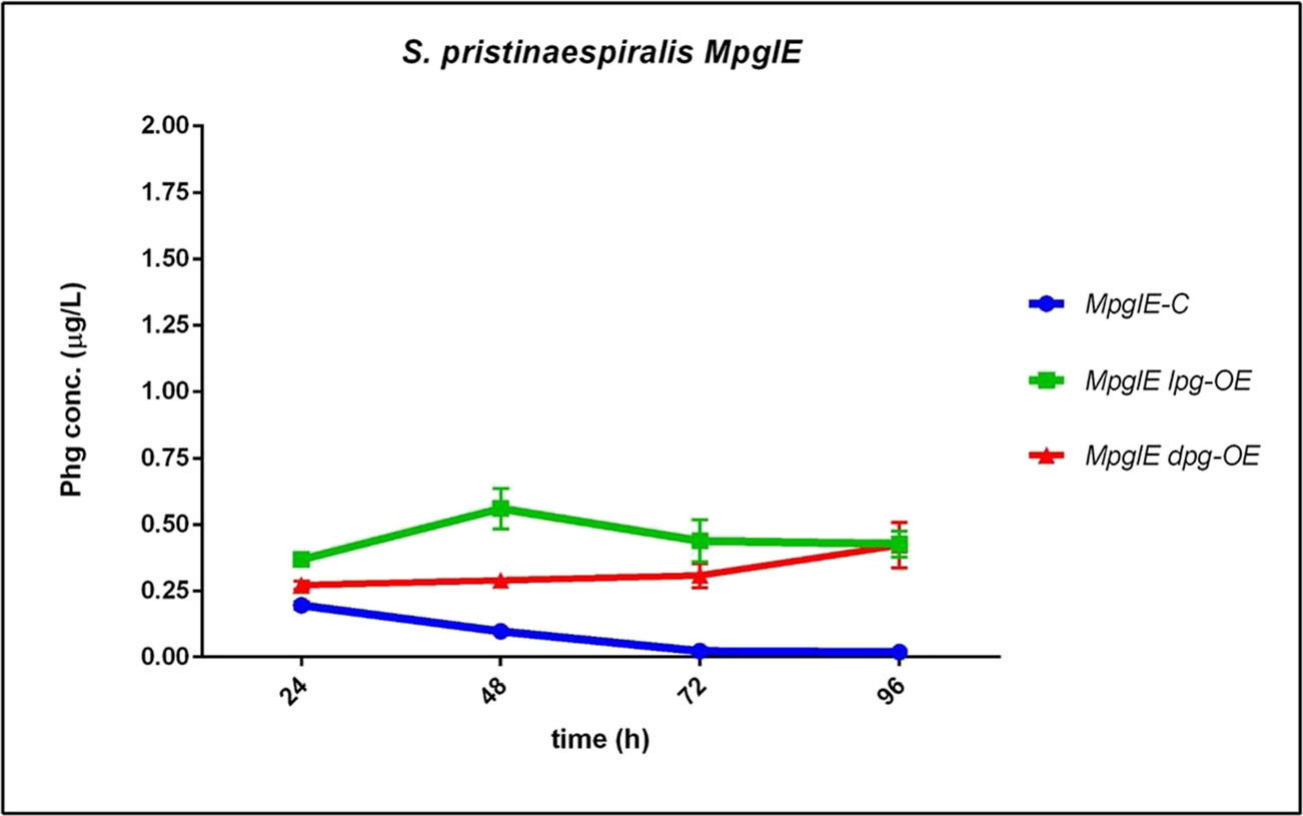
Phg production of *S. pristinaespiralis MpglE* host strains *MpglE lpg-OE* and *MpglE dpg-OE*, (control: *MpglE-C*) grown in HT7T. Phg production was measured at 24, 48, 72, and 96 h. Phg concentration is given in micrograms per liter. Data were obtained from three independent biological replicates

In a recent study, we showed that the L-Phg aminotransferase PglE is responsible for the conversion of phenylglyoxylate to L-Phg in *S. pristinaespiralis* (Osipenkov et al. 2018) (Fig. 2). Deletion of *pglE* leads to an accumulation of phenylglyoxylate (Osipenkov et al. 2018). Due to this increased basal precursor availability, we were interested how the Phg operon expression in the *S. pristinaespiralis pglE* mutant (*MpglE*) would influence production performance. Besides that, inactivation of the native *pglE* gene could deliver a genetic background for the production of enantiopure Phgs in *S. pristinaespiralis*. Thus, the *MpglE* mutant was used as parental strain for the expression of the Phg operons. Strain denomination is similar as reported above and samples were treated as outlined before. HPLC-MS/MS analysis revealed that Phg production in *MpglE* host strains (*MpglE lpg-OE* and *MpglE dpg-OE*) was overall slightly higher than in *S. pristinaespiralis* wild-type-derived strains (Fig. 6 vs Fig. 4d): An improvement was observed for the *MpglE lpg-OE* samples, where a maximal production of 0.56 μg/L Phg at 48 h was measured, which is an increase of 1.8-fold compared with the maximal value of 0.31 μg/L Phg at 24 h in the *SPlpg-OE* sample. For *MpglE dpg-OE* expression samples, no tremendous Phg production improvement was observed (Fig. 6). Therefore, one could speculate that the slightly increased Phg rates in *MpglE lpg-OE* may result from a somehow favorable basal phenylglyoxylate precursor supply.

### Deletion of the PI-NRPS gene *snbDE* significantly improves Phg production in *S. pristinaespiralis*

**Fig. 7:**
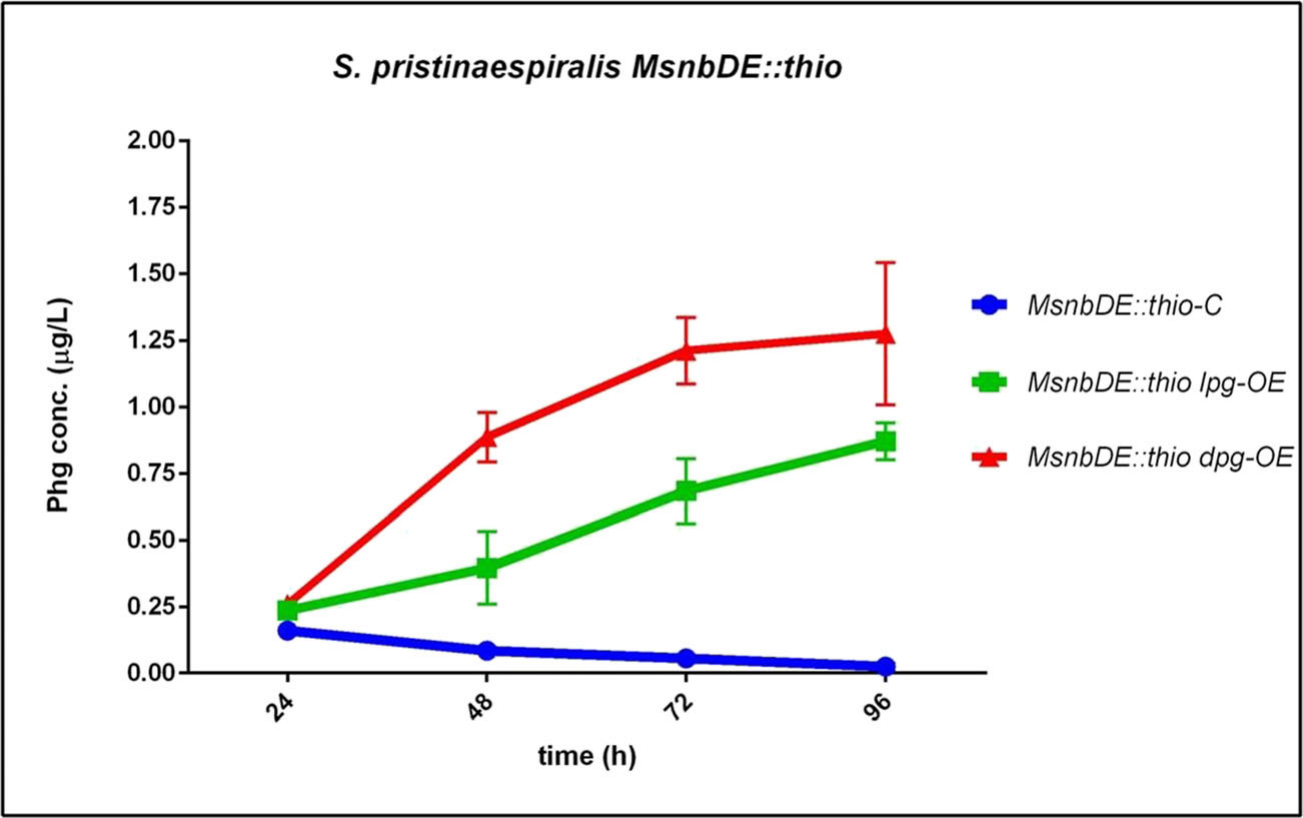
Phg production of *S. pristinaespiralis snbDE* host strains *MsnbDE::thio lpg-OE* and *MsnbDE::thio dpg-OE*, (control: *MsnbDE::thio-C*) grown in HT7T. Phg production was measured at 24, 48, 72, and 96 h. Phg concentration is given in micrograms per liter. Data were obtained from three independent biological replicates

As suggested above, the decrease of Phg in *SPlpg-OE* samples may be due to an incorporation of L-Phg into PI (Fig. 4d). Thus, a strategy to increase Phg production in *S. pristinaespiralis* is to block PI biosynthesis. In order to do that, we inactivated the gene *snbDE* in *S. pristinaespiralis* (Supplementary File), which encodes the PI-specific NRPS module SnbDE that uses L-Phg as a building block for PI biosynthesis (Mast et al. 2011b). The respective mutant *MsnbDE::thio* was used as expression host for the Phg operon expression. The derived host strains *MsnbDE::thio lpg-OE* and *MsnbDE::thio dpg-OE*, as well as the control *MsnbDE::thio-C*, were grown in HT7T and samples were analyzed for Phg production by HPLC-MS/MS. HPLC-MS/MS analysis revealed a maximal Phg production in samples *MsnbDE::thio lpg-OE* (0.87 μg/L) and *MsnbDE::thio dpg-OE* (1.27 μg/L) at 96 h, which was an increase of ~ 3-fold compared with maximal production values in wild-type-derived samples *SPlpg-OE* and *SPdpg-OE* (0.30 μg/L and 0.35 μg/L), respectively (Fig. 7 vs Fig. 4d). Furthermore, it was found that Phg concentration in the *MsnbDE::thio*-derived strains increased continuously, whereas a decrease was observed in the wild-type-derived samples at later time points. Actually, the Phg production profile of the *MsnbDE::thio*-derived strains more resembled the production profile of the *S. lividans* host strains (Fig. 7 vs Fig. 4b). Thus, it can be assumed that Phg production in the *MsnbDE::thio*-derived strains is steadily increasing because Phg is not utilized for PI biosynthesis and thus accumulates, which may also happen in *S. lividans* because this strain does not produce pristinamycin.

### Deletion of the pristinamycin TetR–like regulatory gene *papR5* significantly improves Phg production in *S. pristinaespiralis*

**Fig. 8:**
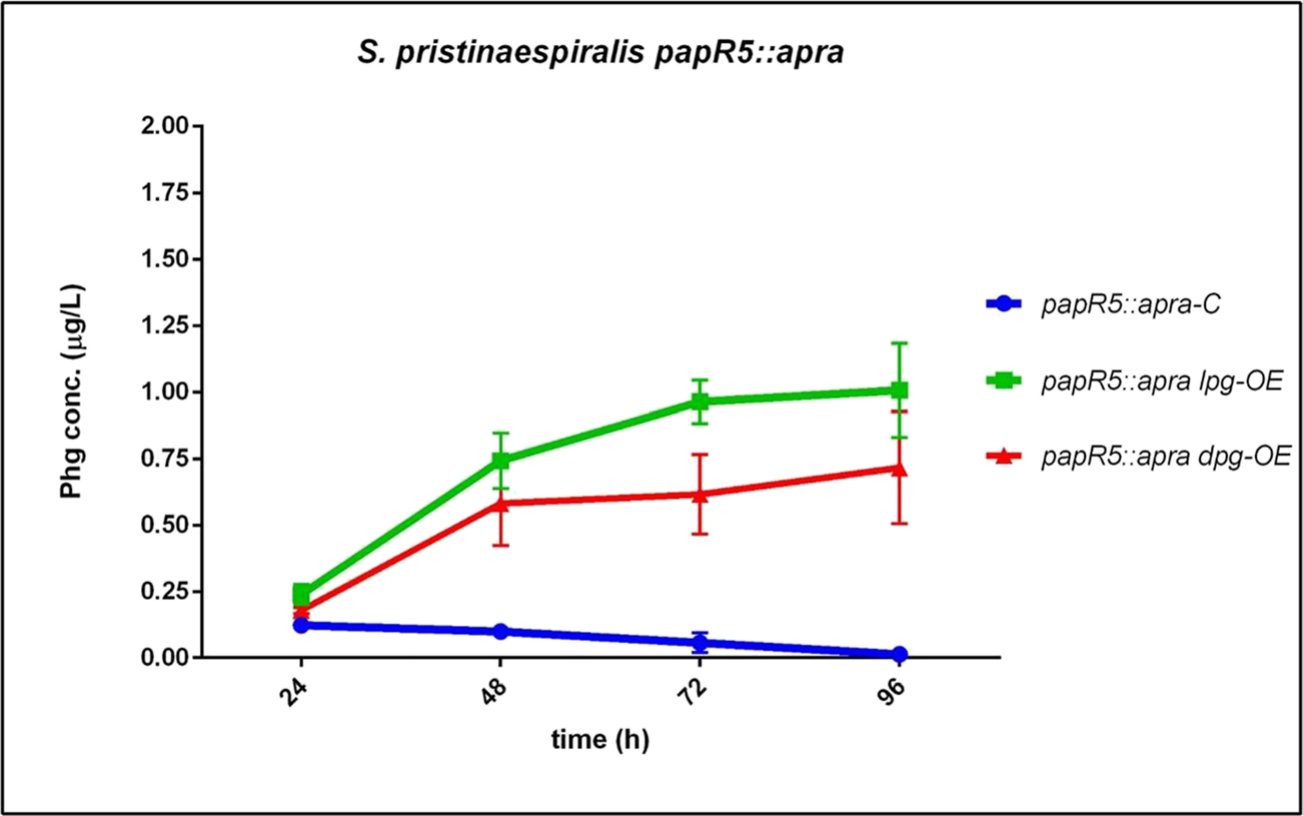
Phg production in the *S. pristinaespiralis papR5* host strains *papR5::apra lpg-OE* and *papR5::apra dpg-OE*, (control: *papR5::apra-C*) grown in HT7T. Phg production was measured at 24, 48, 72, and 96 h. Phg concentration is given in micrograms per liter. Data were obtained from three independent biological replicates

As we had incident that Phg production performance in *S. pristinaespiralis* depends on the pristinamycin biosynthesis capability (see above for *MpglE, MsnbDE* samples), we aimed to further enhance Phg production by using a pristinamycin superproducer as expression host. In a previous study, we showed that the *S. pristinaespiralis* repressor mutant *papR5::apra* produces up to ~ 300% more pristinamycin than the wild-type strain (Mast et al. 2015). Due to this high pristinamycin production capability, we used *papR5::apra* as a host for Phg operon expression. The derived host strains *papR5::apra lpg-OE* and *papR5::apra dpg-OE*, as well as the control strain *papR5::apra-C*, were grown in HT7T and samples were analyzed by HPLC-MS/MS for Phg production. HPLC-MS/MS data revealed that Phg production was significantly increased in *papR5::apra*-derived host strains compared with the wild-type-derived ones: *papR5::apra lpg-OE* and *papR5::apra dpg-OE* produced approximately 3.3-fold and 2-fold, respectively, more Phg than the wild-type-derived expression strains (*papR5::apra lpg-OE*: 1 μg/L; *papR5::apra dpg-OE*: 0.72 μg/L Phg) (Fig. 8 vs Fig. 4d). Phg production was increased especially in the *papR5::apra lpg-OE* host strain, which was also observed for the other Phg precursor–engineered host strains (*SLpaa::thio*, *SPpaa::thio*, and *MpglE*). Thus, Phg-related precursor engineering seems to affect more L-Phg than D-Phg biosynthesis. This might be explained by the less favorable enzymatic properties of HpgAT, as mentioned before. The reason why Phg concentration is stable or even increasing in these expression hosts might be because PI production is oversaturated with Phg precursor and thus Phg would accumulate. Overall, the improvement of Phg production in the *papR5::apra*-derived host strains most likely results from the elevated levels of precursor supply in the course of an increased pristinamycin biosynthesis.

## Discussion

Non-proteinogenic amino acids, such as Phg, are important building blocks and precursors for the synthesis of industrial relevant pharmaceuticals and other fine chemicals. We could show that genetic engineering of suitable target genes in appropriate expression strains allows improvement of Phg production. As expression hosts, we tested different actinomycetal species. This is the first time that Phg production has been accomplished in actinomycetes. Even if Phg production was quite efficient in *S. lividans* expression strains, *S. pristinaespiralis* is preferred as producer host since it offers a broader range of target genes, suitable for genetic engineering to increase production yields. The strongest Phg production improvement was observed for *S. pristinaespiralis* expression strains with an inactivated phenylacetyl-CoA degradation pathway (*SPpaa::thio* strains). Here, production levels were quite high from the beginning on, which might be the result of so far unknown feedback/feedforward control of Phg biosynthesis in *S. pristinaespiralis*. Maximal production of *SPpaa::thio* was measured at ~ 1.6 μg/L Phg, which corresponds to a 4.6-fold increase compared with the production levels of wild-type-derived strains. However, also mutations of specific PI biosynthesis genes (*pglE, snbDE*) and a pristinamycin transcriptional regulator gene (*papR5*) led to an increase of Phg production. Especially for these producer strains it would be interesting to also determine PI production levels in order analyze how they correlate with Phg production profiles. Furthermore, it should be investigated if D-Phg can be used as a building block for PI biosynthesis, which is not known so far. This could be assumed since Phg production was increased in both PI-NRPS deletion host strains, *MsnbDE::thio lpg-OE* and *MsnbDE::thio dpg-OE*, suggesting that both Phg enantiomers are used as PI building blocks*.*

A logic strategy to further increase Phg production is to perform a combinatory genetic engineering approach and inactivate all the above-mentioned genes in *S. pristinaespiralis*. An additional target gene for further production improvement is *papR2*, which encodes a SARP-type transcriptional regulator that is suggested to activate the L-Phg operon in *S. pristinaespiralis* (Mast et al. 2015). Thus, additional overexpression of *papR2* in an engineered *S. pristinaespiralis* Phg superhost could further increase production yields. Due to these multifarious manipulation opportunities, *S. pristinaespiralis* indeed represents a good chassis strain for fermentative Phg production. However, even if production improvement worked out quite well for *S. pristinaespiralis*, the overall product concentrations are still low. Maximal production was ~ 1.6 μg/L Phg, which is still far away from mg production levels previously reported for *E. coli* fermentation (Müller et al. 2006).

In *E. coli*, Phg production was accomplished by expressing an artificial Phg operon, consisting of at least three genes (*hmaS*, *hmo*, *hpgAT*, or *pgat*) from different organisms (*Streptomyces coelicolor*, *Amycolatopsis orientalis*, and *Pseudomonas putida*) in a suitable pathway-engineered *E. coli* strain (Müller et al. 2006; Liu et al. 2014). Thereby, Phg production was optimized to 51.6 mg/g dry cell weight L-Phg (Liu et al. 2014, 2015) and 102 mg/g dry cell weight D-Phg (Müller et al. 2006). In another approach, L-Phg production was accomplished in *E. coli* by co-expression of a leucine dehydrogenase from *Bacillus cereus* (*Bc*LeuDH) and a NAD^+^-dependent mutant formate dehydrogenase from *Candida boidinii* (*Cb*FDH_A10C_), which yielded 28.4 mg/g dry cell weight L-Phg (Liu et al. 2018). However, these values are not comparable with those of our study since production volumes are given in mg/g biomass in the *E. coli* studies but were measured in micrograms per liter of Phg from culture supernatant samples in the present study. Indeed, it was not possible to correlate biomass values (dry cell weight) with Phg production outputs in *Streptomyces* samples due to their irregular growth in liquid cultures, which leads to strong deviations in biomass values. In any way, absolute Phg concentrations for *Streptomyces* samples are hard to determine exactly by HPLC-MS/MS quantification. What we rather would like to depict is that Phg production and optimization can clearly be followed by comparison with control strains. Notably, this is the first time that fermentative Phg production has been reported for actinomycetes. Furthermore, it is important to mention that, unlike the studies in *E. coli*, the precursor supply of phenylpyruvate was not modified in our strains. This is a pivotal target for further studies as usually the aromatic amino acid biosynthesis pathway in bacteria is strictly regulated and limits the precursor supply (Huccetogullari et al. 2019; Lee and Wendisch 2017; Rodriguez et al. 2014; Sprenger 2007). It was shown for *Streptomyces venezuelae* that an improved flux through the shikimate pathway by overexpressing the genes of shikimate kinase (*aroK*) and dehydroquinate synthase (*aroB*) increased the production of the aromatic antibiotic chloramphenicol (Vitayakritsirikul et al. 2016). Overexpression of the gene chorismate synthase (*aroC*) in *Streptomyces tsukubaensis* improved the production of the immunosuppressant tacrolimus (Wang et al. 2017). Furthermore, overexpression of the 3-deoxy-D-arabino-heptulosonate 7-phosphate synthase gene (*dahp*) and the prephenate dehydrogenase gene (*pdh*) in *A. balhimycina* resulted in improved balhimycin production (Thykaer et al. 2010). Thus, it is very likely that Phg amount can be further increased by improving precursor supply from the shikimate pathway. In our study, we could ensure and show that the final reaction, the conversion of phenylpyruvate to Phg, can be improved by deleting the competing reaction for Phg biosynthesis.

*S. pristinaespiralis*-derived Phg operons indeed are interesting molecular entities for future applications. So far, the *pgl* genes from *S. pristinaespiralis* encode the only known natural Phg biosynthesis pathway. One could assume that this pathway is already evolutionary optimized and under appropriate conditions might be more powerful than an artificially assembled pathway harboring genes from different origins. However, this would require further investigations. Overall, the Phg operons represent promising biobricks for Phg-related production processes. A purely fermentative production route has mainly been prevented by the absence of a natural Phg pathway. In this study, we could describe the functionality of the natural L-Phg operon from *S. pristinaespiralis* and its derived D-Phg operon obtained by a synthetic biology approach. The new fermentative Phg production route serves as a basis to replace the environmentally unfriendly industrial Phg production process.

## Electronic supplementary material

ESM 1 (PDF 487 kb)
