# Supplementary material for: Genetic engineering approaches for the fermentative production of phenylglycines
Source: Appl Microbiol Biotechnol. 2020 Feb 20;104(8):3433–44. doi: 10.1007/s00253-020-10447-9 (PMC7089894; doi:10.1007/s00253-020-10447-9)
Supplement: Supplementary file 1 — (PDF 487 kb) [file 253_2020_10447_MOESM1_ESM.pdf]

Supplementary Material

Applied Microbiology and Biotechnology

Genetic engineering approaches for the fermentative production of phenylglycines

David Moosmann[1], Vladislav Mokeev[1], Andreas Kulik[1], Natalie Osipenkov[1], Susann Kocadinc[1], Regina Ort-Winklbauer[1], Franziska Handel[1], Oliver Hennrich[1], Jung-Won Youn[2], Georg A. Sprenger[2] and Yvonne Mast[1,3,4,5]*

[1]Microbiology/Biotechnology, Interfaculty Institute of Microbiology and Infection Medicine, Faculty of Science, University of Tübingen, Auf der Morgenstelle 28, D-72076 Tübingen, Germany

[2]Institute of Microbiology, University Stuttgart, Allmandring 31, D-70569 Stuttgart, Germany

[3]German Center for Infection Research (DZIF), Partner Site Tübingen, Tübingen, Germany

[4]Department „Bioresources for Bioeconomy and Health Research“, Leibniz Institute DSMZ-German Culture Collection for Microorganisms and Cell Cultures, 38124 Braunschweig, Germany

[5]Institute for Microbiology, Technical University of Braunschweig, 38106 Braunschweig, Germany

Phone: +49(0)531 2616 358, Telefax: +49(0)531 2616 418

Electronic mail address: yvonne.mast@dsmz.de

ORCID ID: Yvonne Mast (0000-0003-3099-5777)

*Corresponding author: Prof. Dr. Yvonne Mast, Phone: +49(0)531 2616 358, e-mail yvonne.mast@dsmz.de

Table S1: Bacterial strains, plasmids, and oligonucleotides.

Bacterial strains, and plasmids	Relevant properties	Source reference
E. coli		
XL1 Blue	*rec*A1 *end* A1 *gyr*A96 *thi*-1 *hsd*R17 *sup*E44 *rel*A1 *lac* [F', *pro*AB *lac1*^{q} *Z*Δ M1*5* Tn*10*(*tet*^{r})]	[1]
Rosetta 2 (DE3)/pLysS	F-, *ompT, hsd*SB, (r_{B} m_{B-}), *gal, dcm,* (DE3), pLys SRARE2, (cm^{R})	Novagen
E. coli pYMpglE	*E. coli* Rosetta 2 (DE3)/pLysS, pYMpglE	This work
S. pristinaespiralis		
Pr11	pristinamycin producing strain / wild-type natural isolate of *S. pristinaespiralis* ATCC 25486	Aventis Pharma
SPpaa::thio	gene deletion of *paaABCDE*, thio^{R}	This work
MpglE	gene interruption of *pglE*, *aac(3)IV*	[2]
MsnbDE::thio	gene interruption of *snbDE*, thio^{R}	This work
papR5::apra	gene interruption of *papR5*, *aac(3)IV*	[3]
SP-C	*S. pristinaespiralis*, pRM4	This work
SPlpg-OE	*S. pristinaespiralis*, pYM/lpg	This work
SPdpg-OE	*S. pristinaespiralis,* pYM/dpg	This work
SPpaa::thio-C	*SPpaa::thio* with pRM4	This work
SPpaa::thio lpg-OE	*SPpaa::thio* with pYM/lpg	This work
SPpaa::thio dpg-OE	*SPpaa::thio* with pYM/dpg	This work
MpglE-C	*MpglE* with pYMT	This work
MpglE lpg-OE	*MpglE* with pYMT/lpg	This work
MpglE dpg-OE	*MpglE* with pYMT/dpg	This work
MsnbDE::thio-C	*MsnbDE::thio* with pRM4	This work
MsnbDE::thio lpg-OE	*MsnbDE::thio* with pYM/lpg	This work
MsnbDE::thio dpg-OE	*MsnbDE::thio* with pYM/dpg	This work
papR5::apra-C	*papR5::apra* with pYMT	This work
papR5::apra lpg-OE	*papR5::apra* with pYM/lpg	This work
papR5::apra dpg-OE	*papR5::apra* with pYM/lpg	This work
S. lividans		
T7	*tsr*, T7-RNA-polymerase gene	[4]
SL-C	*S. lividans* T7, pRM4	This work
SLlpg-OE	*S. lividans* T7, pYM/lpg	This work
SLdpg-OE	*S. lividans* T7, pYM/dpg	This work
TK23	Wild-type	[5]
SLpaa::thio	*S. lividans* TK23, gene deletion of *paaABCDE*, thio^{R}	This work

SLpaa::thio-C	*SLpaa::thio* with pRM4	This work
SLpaa::thio lpg-OE	*SLpaa::thio* with pYM/lpg	This work
SLpaa::thio dpg-OE	*SLpaa::thio* with pYM/dpg	This work
S. albus		
S. albus J1074	Wild-type	[6]
SA-C	*S. albus*, pRM4	This work
SAlpg-OE	*S. albus*, pYM/lpg	This work
SAdpg-OE	*S. albus*, pYM/dpg	This work
A. balhimycina		
DSM5908	Wild-type	[7]
AB-C	*A. balhimycina*, pRM4	This work
ABlpg-OE	*A. balhimycina*, pYM/lpg	This work
ABdpg-OE	*A. balhimycina*, pYM/dpg	This work
R. jostii		
RHA1	Wild-type; Nal^{R}	[8]
RJ-C	*R. jostii,* pRM4	This work
RJlpg-OE	*R. jostii*, pYM/lpg	This work
RJdpgD-OE	*R. jostii*, pYM/dpg	This work
**Cosmids and plasmids**		
pYJM1	cosmid carrying the *pglA-E* gene, *aac(3)IV*	[2]
pDrive	*lacZ'*-complementation system, ampicillin and kanamycin resistance, multiple cloning site	Qiagen
pRM4	pSET152*ermEp** derivate with artificial RBS (ΦC31 integration vector with PermE, Apra^{R})	[9]
pK18	pUC derivative, *aphII*, *lac*Z'- complementation system	[10]
pEH13	pUC21 derivative carrying the 1.8 kb apramycin resistance cassette (*apr*^{R})	[11]
pDrive-thio	pDrive, thio^{R}	[12]
pYM/lpg	pRM4 derivative with *lpg* operon	This work
pYM/dpg	pRM4 derivative with *dpg* operon	This work
pYMT	pRM4 derivative with thio^{R}	This work
pYMT/lpg	pRM4 derivative with thio^{R}, *lpg* operon	This work
pYMT/dpg	pRM4 derivative with thio^{R}, *dpg* operon	This work
pYM/snbDE	pK18 derivative, *aph*II, *thio*^{R}, *lac*Z'*α*, *snbDEab*	This work
pYM/SPpaa	pK18 derivative, *aph*II, *thio*^{R}, *lac*Z'*α*, *SPpaa_ab*	This work
pYM/SLpaa	pK18 derivative, *aph*II, *thio*^{R}, *lac*Z'*α*, *SLpaa_ab*	This work

Primer	Primer sequence (5’-3’)	Temp.
**Primer for expression studies/cloning procedures**		
lpgfw	ATCATATGCGCACACCGACCCTCG	66°C
lpgrv	ATAAGCTTGCGCGGTGTCCTCGGAC	68°C
pglDfus1	TGCGACTGCTGTGCTTC	55°C
pglDfus2	CATAATCGCTATAAATAGACATTATATCGGTCTCCCGGTGTTC	70°C
hpgATfus1	GAACACCGGGAGACCGATATAATGTCTATTATAGCGATTATG	65°C
hpgATfus2	ATAAGCTTTCATATTCCAGACAGATGTG	61°C
**Primer for mutant construction**		
snbDEm1	CGTTCGAGGGTGCTGATGAG	58°C
snbDEm2	ATGATATCGTGCGCACACCGACCCTCGC	67°C
snbDEm3	ATGATATCATGGGGACACCTTCCACGCG	64°C
snbDEm4	GTCTCCTGGCGCATCCTG	59°C
SLpaam1	GAATTCTCCACCCGCACCGTCATGTG	64°C
SLpaam2	TCTAGAATGAGCAGCGAGCCGACGAC	64°C
SLpaam3	TCTAGAACTACGACAGCTGACCGGGT	62°C
SLpaam4	AAGCTTACAACCCCGCCTAGGTGTGT	64°C
SPpaam1	GAATTCTGTCGCGGTCCTTGTTGTCG	62°C
SPpaam2	TCTAGAATCCGGACTCCCCTACCGAC	62°C
SPpaam3	TCTAGAACAAGCCCCTATCGACACCG	61°C
SPpaam4	AAGCTTATGGCCCGCAGATCGATGTA	62°C
**Primer for mutant verification**		
thio1	CGTTGGTGATTGCCGGTCAG	59°C
thio2	GGCGATGCCGAATGTCTTGG	59°C
**Primer for amplification of cDNA (RT-PCR)**		
RTsnbDE/pglAfw	ATCGAGGAGCACCACCTGGA	61°C
RTsnbDE/pglArv	TCCTTGGTGAACTCGCCGGT	61°C
RTpglA/Bfw	TACCTCGCCCGGTACGCCTA	64°C
RTpglA/Brv	TGACCATGGCCCGGTACAGC	64°C
RTpglB/Cfw	AGATGTTCCAGCACGTCTAC	57°C
RTpglB/Crv	AAGACGAGGGTGTTCTCGTC	59°C
RTpglC/Dfw	ACCGGCTTCGACGTGCCCTA	64°C
RTpglC/Drv	TGCGGGAAGCACAGCAGTCG	64°C
RTpglD/Efw	AACACCGGGAGACCGATATG	59°C
RTpglD/Erv	TTGAGGAGGGTCATCGAGGT	59°C
hrdBfw	TGGTCGAGGTCATCAACAAG	50°C

hrdBrv	TGGACCTCGATGACCTTCTC	52°C

restriction sites are underlined

Cloning charts of Phg expression constructs

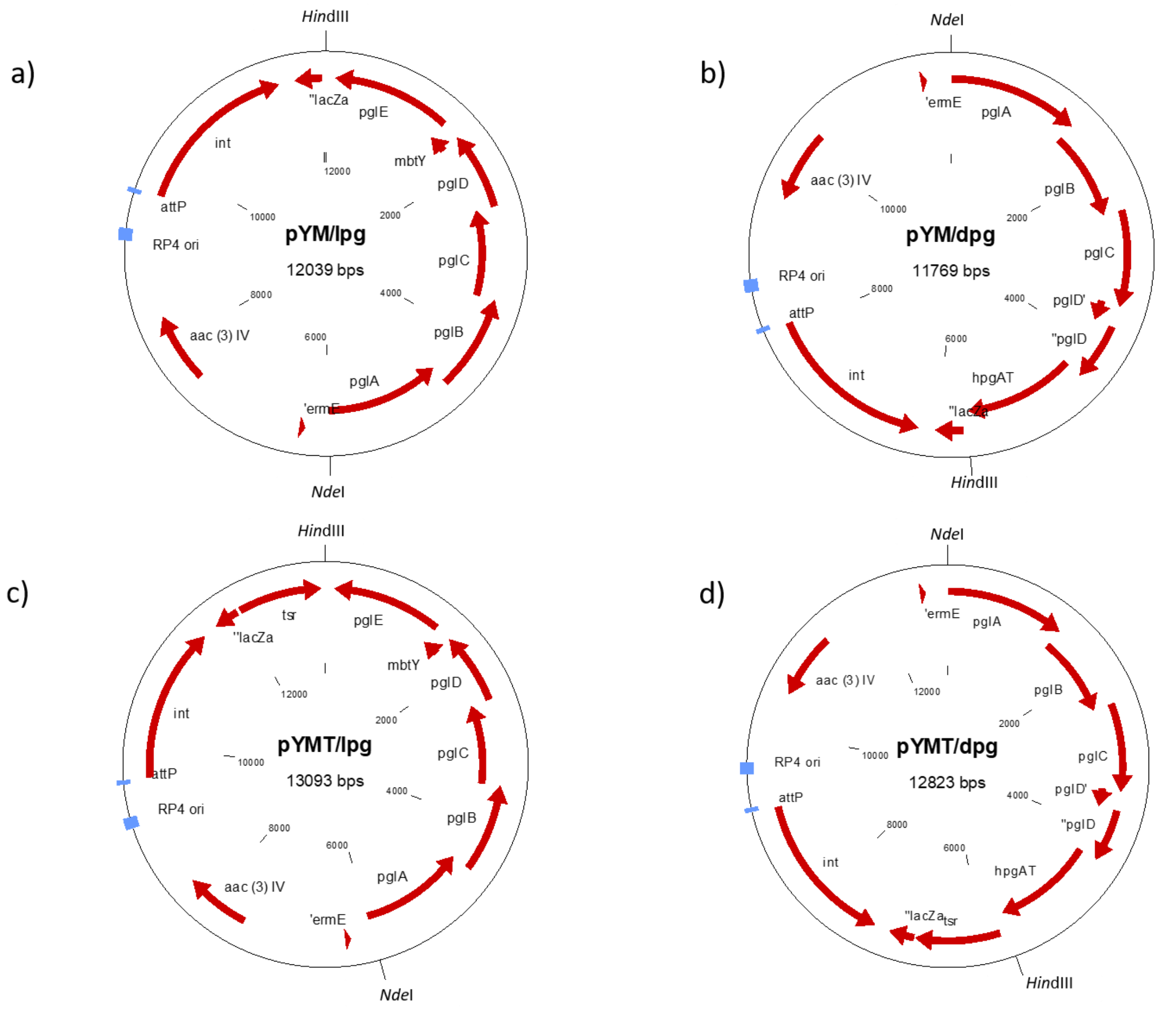

Fig. S1: Plasmid charts of pYM/lpg (a), pYM/dpg (b), pYMT/lpg (c), and pYMT/dpg (d).

Statistical data on Phg production in HT7T and R5 medium

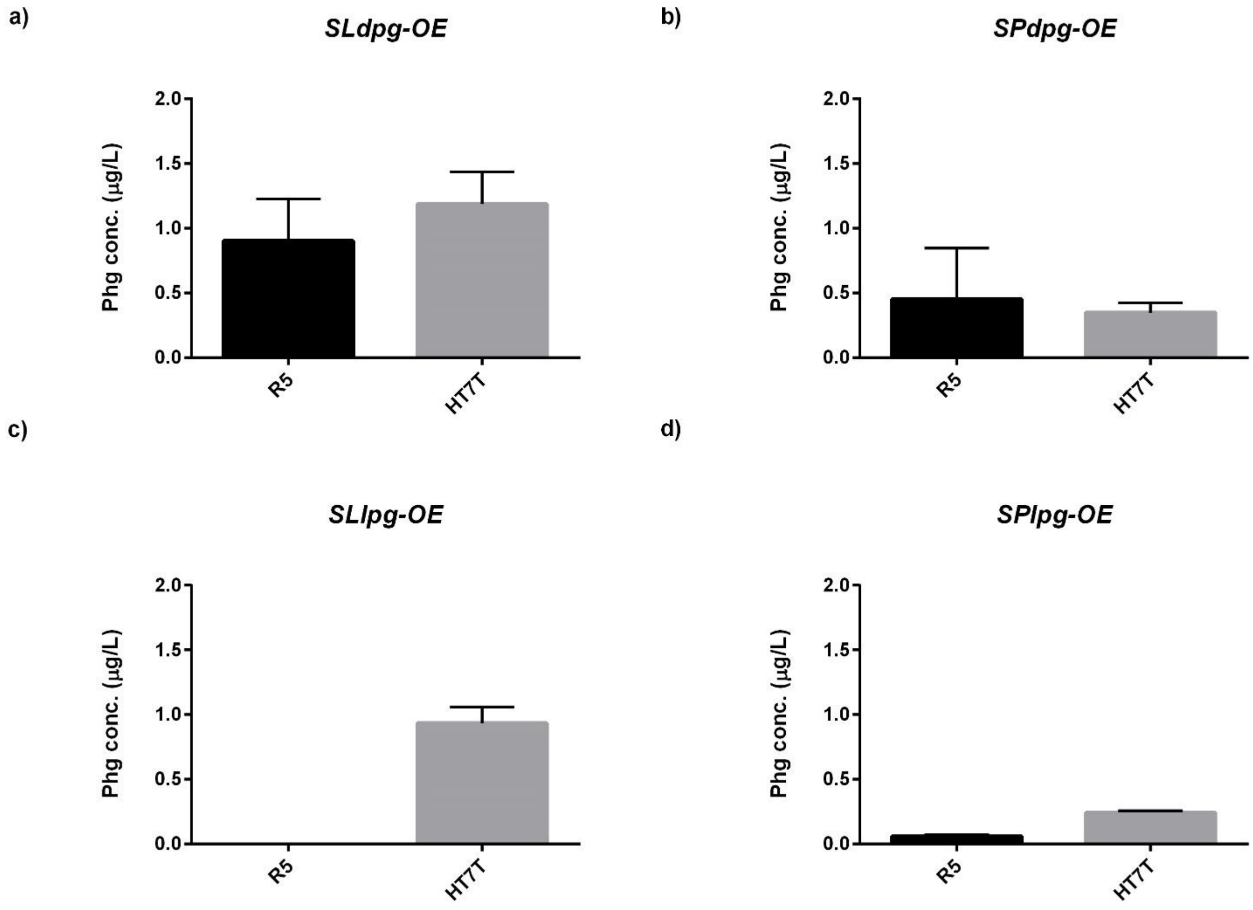

Figure S2: Average Phg concentration for tested expression strains at the respective time of highest production in R5 media compared to HT7T media. Unpaired t-test was applied for calculating significance of data. The significance level of $p \leq 0.05$ was used for all data analyses in this study. Confidence interval was 95%. Unpaired *t*-tests show a significant statistical difference for *SLlpg-OE* (c) and *SPlpg-OE* (d) (p-values of 0.0002 and <0.0001, respectively) but not for *SLdpg-OE* (a) and *SPdpg-OE* (b) (p-values of 0.2943 and 0.6750, respectively).

Construction of mutants *MsnbDE::thio, SPpaa::thio*, and *SLpaa::thio*

For construction of the MsnbDE::thio mutant, up- and downstream fragments of *snbDE* were amplified by PCR using *S. pristinaespiralis* Pr11 genomic DNA and primers listed in Additional File 1. For amplification of the downstream fragment, primer pairs labeled as m1/m2 were used that added an artificial *Eco*RV restriction site to the 5' end, resulting in the amplificate *snbDEa* (1.6 kb). For amplification of the upstream fragment, primer pairs labeled as m3/m4 were used that added an artificial *Eco*RV restriction site to the 3' end, resulting in the amplificate *snbDEb* (1.1 kb). The *snbDEa* and *snbDEb* fragments were subcloned into the *E. coli* vector pDrive, resulting in the constructs pDrive/*snbDEa* and pDrive/*snbDEb,* respectively*. snbDEa* was isolated from pDrive/*snbDEa* as a *Xba*I/*Bam*HI fragment and ligated into the *Xba*I/*Bam*HI-restricted *E. coli* vector pK18, resulting in the construct pK18/*snbDEa*. *snbDEb* was excised as a *Hin*dIII/*Eco*RV fragment from pDrive/*snbDEb* and ligated into the *Hin*dIII/*Eco*RV site of pK18/*snbDEa*, which resulted in the constructs pK18/snbDE*'*. A 1.1 kb thiostrepton resistance cassette (*thio*^{R}) was isolated as a *SnaBI*/*AleI* fragment from pDrive-thio and cloned into the *Eco*RV restriction site between the *snbDEa* and *snbDEb* fragment of pK18/*snbDE'*, resulting in the mutational constructs pYM/*snbDE* in which the *snbDE* gene is inactivated by the insertion of the *thio*^{R} cassette.

For construction of the SPpaa::thio and SLpaa::thio mutants, ~1-1.5 kb fragments up- and downstream of the *paaABCDE* gene regions from *S. pristinaespiralis* (SSDG_0039-SSDG_0043) and *S. lividans* (SSPG_00371-SSPG_00376) were amplified by PCR using genomic DNA from *S. pristinaespiralis* Pr11 and *S. lividans* TK23, respectively, and primers listed in Additional File 1. For cloning reasons *S. lividans* TK23 was used as parental strain for *paa* mutagenesis since *S. lividans* T7 is

thiostrepton resistant. For amplification of the downstream fragments, primer pairs labeled as m1/m2 were used that added an artificial *XbaI* restriction site to the 5' end, resulting in amplificates *SPpaa_a* (1.0 kb), and *SLpaa_a* (1.2 kb), respectively. For the amplification of the upstream fragments, primer pairs labeled as m3/m4 were used that added an artificial *XbaI* restriction site to the 3' end, resulting in the amplificates *SPpaa_b* (1.1 kb), and *SLpaa_b* (1.3 kb), respectively. All fragments were subcloned into the *E. coli* vector pDrive, resulting in the constructs pDrive/*SPpaa_a, b* and pDrive/*SLpaa_a, b,* respectively. All "*a*" fragments were isolated from the respective pDrive/*paa*_a constructs as *Eco*RI/*Xba*I fragments, whereas all "*b*" fragments were isolated from the respective pDrive/*paa*_b constructs as *Xba*I/*Hin*dIII fragments. The "a" and "b" fragments were ligated into the *Eco*RI/*Hin*dIII-restricted *E. coli* vector pK18, resulting in the constructs pK18/*SPpaa'* and pK18/*SLpaa',* respectively. A 1.1 kb thiostrepton resistance cassette (*thio*^{R}) was isolated as a *Xba*I fragment from pDrive-thio and was cloned into the *Xba*I restriction site between fragment "*a*" and "*b*" of the respective pK18/*SPpaa'* and pK18/*SLpaa'* derivatives, resulting in the mutational constructs pYM/*SPpaa* and pYM/*SLpaa,* respectively. Thereby the *paaABCDE* gene region was inactivated by the insertion of the *thio*^{R} cassette.

The mutational constructs were transferred into *S. pristinaespiralis* Pr11 or *S. lividans* TK23, respectively, by protoplast transformation, followed by selection for thiostrepton-resistant and kanamycin-sensitive transformants, resulting in the mutants *MsnbDE::thio, SPpaa::thio,* and *SLpaa::thio*, respectively. Mutants were confirmed by PCR, exemplary shown for here by amplification of thiostrepton resistance cassette *thio*^{R} (Fig. S3).

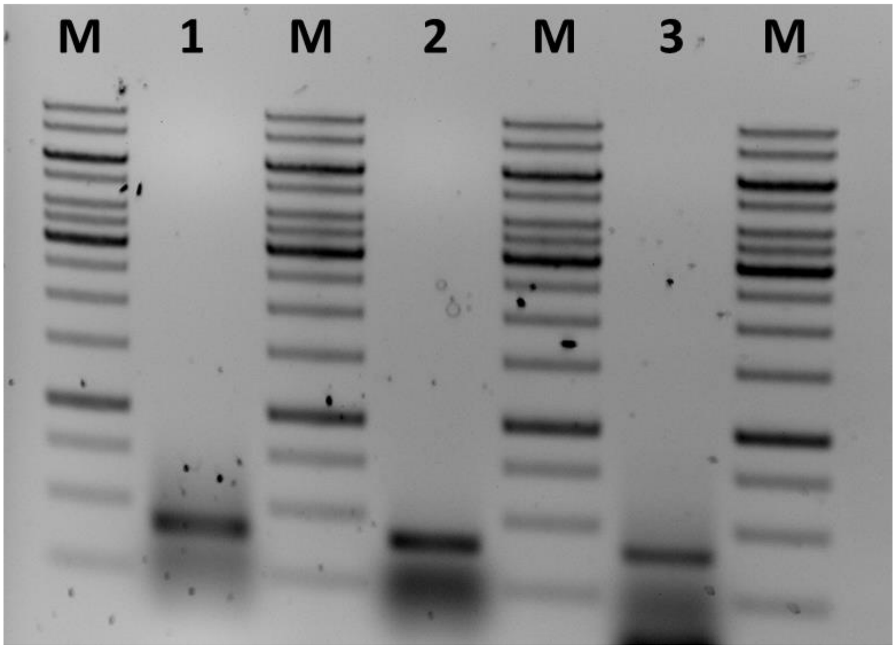

Figure S3: Verification of the 398 bp *thio*^{R} amplificates in a 1% agarose gel. 5 µl of the GeneRuler 1-kb ladder (Fermentas) was used as marker (M). Samples were obtained from PCR with genomic DNA of *MsnbDE::thio* (1), *SPpaa::thio* (2), and *SLpaa::thio* (3) and primer pair thio1/2.

Construction of mutant-derived Phg expression strains

For Phg expression in thiostrepton-resistant mutants (*MsnbDE::thio, SPpaa::thio* and *SLpaa::thio*) the expression constructs pYM/*lpg* and pYM/*dpg*, as well as the empty plasmid pRM4 were each transferred to the respective mutants via protoplast transformation. For expression of the Phg operons in apramycin-resistant mutants (*MpglE* and and *papR5::apra*) the expression constructs pYMT/*lpg* and pYMT/*dpg*, as well as the empty plasmid pYMT were used for protoplast transformation of the respective mutants. Transformants were obtained via selection for apramycin- and thiostrepton-resistant colonies.

Reference

1. Bullock, W O1. Bullock WO, Fernandez JM SJM. XL1-Blue: A high efficiency plasmid transforming *recA Escherichia coli* strain with beta-galactosidase selection. Biotechniques. 1987;5:376–9.

2. Mast YJ, Wohlleben W, Schinko E. Identification and functional characterization of phenylglycine biosynthetic genes involved in pristinamycin biosynthesis in *Streptomyces pristinaespiralis*. J Biotechnol. 2011;155:63–7.

3. Mast Y, Guezguez J, Handel F, Schinko E. A complex signaling cascade governs pristinamycin biosynthesis in *Streptomyces pristinaespiralis*. Appl Environ Microbiol. 2015;81:6621–36.

4. Sambrook J, Fritsch EF, Maniatis T. Molecular Cloning: A Laboratory Manual. 2nd ed. New York: Cold Spring Harbor laboratory press; 1989.

5. Hopwood DA, Bibb MJ, Chater KF, Kieser T, Bruton CJ, Kieser HM, et al. Genetic manipulation of Streptomyces: A laboratory manual. Norwich, UK: John Innes Foundation; 1986.

6. Chater KF, Wilde LC. *Streptomyces albus* G mutants defective in the SalGI restriction-modification system. J Gen Microbiol. 1980;116:323–34.

7. Nadkarni SR, Patel M V, Chatterjee S, Vijayakumar EK, Desikan KR, Blumbach J, et al. Balhimycin, a new glycopeptide antibiotic produced by *Amycolatopsis* sp. Y-86,21022 - Taxonomy, production, isolation and biological activity. J Antibiot. 1994;47:334–41.

8. Seto M, Kimbara K, Shimura M, Hatta T, Fukuda M, Yano K. A novel transformation of polychlorinated biphenyls by *Rhodococcus sp.* strain RHA1. Appl Environ Microbiol. 1995;61:3353–8.

9. Menges R, Muth G, Wohlleben W, Stegmann E. The ABC transporter Tba of *Amycolatopsis balhimycina* is required for efficient export of the glycopeptide antibiotic balhimycin. Appl Microbiol Biotechnol. 2007;77:125–34.

10. Pridmore RD. New and versatile cloning vectors with kanamycin-resistance marker. Gene. 1987;56:309–12.

11. Heinzelmann E, Kienzlen G, Kaspar S, Recktenwald J, Wohlleben W, Schwartz D. The Phosphinomethylmalate Isomerase Gene *pmi*, Encoding an Aconitase-Like Enzyme, Is Involved in the Synthesis of Phosphinothricin Tripeptide in *Streptomyces viridochromogenes*. Appl Environ Microbiol. 2001;67:3603–9.

12. Pavlidou M, Pross EK, Musiol EM, Kulik A, Wohlleben W, Weber T. The phosphopantetheinyl transferase KirP activates the ACP and PCP domains of the kirromycin NRPS/PKS of *Streptomyces collinus* Tü 365. FEMS Microbiol. Lett. 2011;319:26–33.
